# 91. A journey between the ologists: is it lupus?

**DOI:** 10.1093/rap/rky034.054

**Published:** 2018-09-20

**Authors:** Aicha Bouraoui, Rifat Mazumder, Philip Stapleton, Bhaskar Dasgupta

**Affiliations:** Rheumatology, Southend Hospital, Southend, UNITED KINGDOM

**Introduction:** The diagnosis of lupus can be easily made in patients whose symptoms fit the diagnostic criteria, however it can be sometimes challenging particularly when patients presents atypically, in which cases rheumatologists need to differentiate between real disease versus other differentials of lupus-like syndromes. Herein we describe a challenging case of patients who was referred to rheumatology with lupus like syndrome, and outline the lesson drawn from this case.

**Case description:** This is a case of 49 year old lady. mother of two, Usually fit and well, referred to rheumatology in November 2017 with six months history of: arthralgia, myalgia and constitutional symptoms. She also had history of iron deficiency aneamia which was thought secondary to polyp which was removed. Blood tests orgnaised by GP showed raised CRP of 35, RF: 39 and normocytic anaemia with Hb of 90. During rheumatology review, the patient described an episode of viral illness and subsequent symptoms of tiredness, lethargy, arthralgia, night sweats/ hot flashes. she denied any skin rashes/mouth ulcers/hair loss/Raynaud's. She described finger nail changes which were not present during the clinic consultation. She stopped having periods in April 2017. Her symptoms were initially attributed to peri menopausal syndrome. physical examination was unremarkable except mild ankle swelling. Blood results showed raised CRP: 35, normocytic anaemia, leucopenia og 5 and lymphopenia of 0.7. Blood tests showed raised RF: 39, negative ANA, CCP, unremarkable serum electrophoresis, low C3: 0.7. C4<10, pancytopenia with platelet 82. patient was started on prednisolone 15 mg, and advised to seek urgent medical review if she felt unwell. In December 2017 she presented to ED generally unwell with worsening constitutional symptoms and drenching night sweats. Purpuric rash was see on both lower limbs. She had hair loss, no nail changes, no meningism or stigmata of infective endocarditis. Bloods showed worsening pancytopenia with Hb: 87, WCC: 3.9 N:3.04, Plt: 82, CRP: 46. Low c3 (0.70),c4 (<0.10). Normal U-E, LFTs persistently negative serology including ANA negative. Her urine dip was positive for bloods and protein with high urine PCR of 40. Lungs and pleural spaces were clear on chest x-ray. Infection screen including Heps serology, HIV: negative. A CT scan was organised to rule out mitotic lesions and showed mild pericardial effusion and bulky spleen with 24 x 15 mm ill-defined hypodense area at posterior border, possibly suggestive of small infarction. A small cystic lesion in the left adnexa was noted. No lymphadenopathy were noted. In view of pancytopenia, and ongoing constitutional symptoms we sought an inpatient haematology review which concluded that it is unlikley heamtological malignancy. The case was also discussed in hematology MDT with CT scan review. The probable working diagnosis at that point was vasculitis and to exclude cryoglobulineamia. The patient was discharged on 30mg of prednisolone. In January 2018 the patient was seen by haematology and felt reassured following the outocmnes of hematology MDT. She described feeling much better on 30mg of prednisolone. Constitutional symptoms were resolved and she started to put on weight. Physically she had mild vasculitic rash on both lower limbs with no splinters. She also had symptoms suggestive of peripheral neuropathy confirmed on NCS. Her urine dip remained positive for protein and bloods. Blood tests showed persistent anaemia, worsening lymphopenia (0.36), high CRP and persistently low c3 (0.40),c4 (<0.10). Following discussion with the patient we planned to reduce prednisolone as no clear diagnosis of lupus, to await cryglobulenimia results and to organise an urgent TVUSS. The latter showed suspicious ovarian cyst hence an urgent gynaecology MDT referral was made and outcomes were to organise laraparoscopy for biopsy +/- surgery. February 2018: on reducing prednisolone and feeling generally unwell with relapse of constitutional symptoms, she became acutely unwell and was admitted with sepsis and AKI required ITU stay. CXR showed extensive air space shadowing with air bronchogram in the right mid and upper zones as well as in the left mid and lower zones - suggestive of infection.

lood cultures: strep pneumonia, urine C/S: no growth. Bloods: Hb: 62, WCC: 29.3, N: 26.3, L: 0.5 , CRP: 417, LDH: 835, Creat: 277,Urea: 22.4, APTT: 71.3, UTP:0.11, UCR:2.9. An echo cardiogram showed severe MR with vegetations. Hence she was treated for infective endocarditis. Cryoglobulins samples show a cryoprecipitating IgM lambda paraprotein with positive rheumatoid factor (29) and low C4 <0.10) concentration. The result would be consistent with Type II cryoglobulinaemia. She required ITU admission with IV antibiotics, inotropes support, haemofiltration. She was also reviewed by haematology and started on IV methylprednisolone. She unfortunately developed heart failure and valve replacement was deemed necessary. She finished her six week course of antibiotics for IE and she is currently under haematologyn for further management of lymphoma.

**Discussion:** This case raises number of questions at clinical and whole system levels. The first question: does sero-negative lupus exist? In this case, the patient fits both ACR diagnostic criteria, however she has been persistently ANA negative which made it challenging to consider the diagnosis of lupus and assess its activity.

According to ACR revised criteria 1997: at least 4/11 criteria, compared to àSLICC criteria (2012): either the biopsy-proven lupus nephritis in the presence of ANA or anti-dsDNA as a stand alone criterion; or four criteria with at least one of the clinical and one of the immunologic/ANA criteria. In the case of our patient, the only immunological tests which remained Patrice and suggestive of lupus activity is low c3/c4, but this marker is not specific of lupus and could be associated to other conditions such as haematological malignancies and infections. The second questions is with regards to steroid responsiveness: is it auto immune marker? We tend to use steroid responsiveness frequently in clinical practice in rheumatology. However, many conditions can respond to steroids including malignancies and infections such as subacute infective endocarditis both of which our patient had. Thirdly , this case also highlights the dilemma of specialism: could stronger interdisciplinary ties in complex cases help to achieve better outcomes for patients? How much would the system help to support this process in an NHS struggling with lack of resources and facing high demands? Are there any other ways of work between specialists and generalists? How much are we keeping our generalist hit as we develop our specialist interest? Going back to this case, should we have had closer links with haematologists ? There are number of haematological conditions encountered in rheumatology such as haemolytic anaemia, macrocytosis, pancytpenia, MAS etc. We believe that stronger interdisciplinary relations could be beneficial not only to patients but also in terms of promoting interdisciplinary learning.

**Key Learning Points:** Seronegative lupus is very rare and we need to exclude other differentials before considering the diagnosis. There a number of conditions which are steroid-responsive. Hence, starting steroids without clear diagnosis can mask delay in patient management and increase risk of complications. In multisystem conditions, frequently encountered in rheumatology, we need stronger interdisciplinary relationships to improve values of care for patients and enhance interdisciplinary learning.

**Disclosure: A. Bouraoui:** None. **R. Mazumder:** None. **P. Stapleton:** None. **B. Dasgupta:** None.

